# Postural stability at activation and deactivation of the cochlear implant in adolescents with late lateral implantations: a quasi-experiment

**DOI:** 10.1186/s13102-024-00950-1

**Published:** 2024-07-21

**Authors:** Anna Zwierzchowska, Eliza Gaweł, Agata Krużyńska, Kajetan J. Słomka, Grzegorz Juras

**Affiliations:** 1 Institute of Sport Sciences, Academy of Physical Education in Katowice, Katowice, 40-065 Poland; 2 The School and preschool complex for deaf and hard of hearing children in Katowice, Katowice, 40-126 Poland

**Keywords:** Postural control, Hearing loss, Vestibular disorders, Deafness, Auditory apparatus

## Abstract

**Background:**

Cochlear implantation (CI) surgery has become a prevalent method of hearing rehabilitation, since it has been acknowledged that it impacts effectively on the vestibular system. However, there is still no consensus among clinicians on the most appropriate age and area (lateral/bilateral) of CI surgery in terms of postural control. The present study aimed to assess the postural control in late lateral CI adolescents with different visual (eyes opened(EO)/eyes closed(EC)) and auditory (CI activated/deactivated) conditions and to build a theoretical model of postural control based on sensual compensatory mechanisms that are predominant in late CI individuals. It was hypothesized that kinesthetic sensation and exteroceptors of the superficial sensation are critical for neuromuscular control after late CI.

**Methods:**

A quasi-experimental study protocol was used in this study to assess the postural stability performance in the studied adolescents with different visual and auditory perceptions. 27 adolescent students with hearing loss participated in the study. A force plate (Accu Gait AMTI) with computer software (NetForce) was used in the study to assess the postural stability with four different conditions(EO)/EC), CI activated/deactivated).

**Results:**

vCOP was found to have a significant growing tendency within the conditions of CI activated/deactivated.No statistically significant relationships were noted between the range of the displacement of feet pressure (Area) and both the visual and auditory conditions. Hearing loss etiology was statistically significantly related to the values of vCOP, within the conditions of EO, CI activated/deactivated (*p* < 0.01), what did not occure with the condition of EC (*p* > 0.05). Neuromuscular control with the condition of EC x CI deactivated was found to be based on the kinesthetic-tactual compensatory model.

**Conclusions:**

Kinesthetic sensation and exteroceptors of the superficial sensation seem to be the predominant source of information to maintain postural control in late CI adolescents, regardless of the visual and auditory conditions. The etiology of hearing loss (congenital/acquired) can be a predictor of the values of the vCOP. In order to improve neuromuscular control in this population, it is recommended that the patients perform physical activity tasks, especially to develop core muscles, based on direct stimulation and rotational stability.

**Supplementary Information:**

The online version contains supplementary material available at 10.1186/s13102-024-00950-1.

## Background

In the last decade, cochlear implantation (CI) surgery has become a more prevalent method of hearing rehabilitation since it has been acknowledged that it impacts effectively on the vestibular system both in deaf newborns and older adults with progressive sensorineural hearing loss, enabling them to properly stimulate the auditory system [1]. However, some studies indicated potential risks for the vestibular system related to CI surgery [2, 3], mainly due to an intrinsic intervention that includes inserting an electrode into the cochlea [4]. Nevertheless, the proper function of the vestibular system is known to be the key factor not only for receiving auditory stimulation but also for maintaining gaze stabilization and postural control of the human body [5]. Therefore, the role of CI surgery seems to be of predominant importance for people with deafness.

Even though the benefits of CI are well known, there is still no consensus among clinicians on the most appropriate age and area (lateral/bilateral) of CI surgery. For instance, some studies suggest that bilateral CI can result in a decrease in postural balance compared to lateral implantation [6, 7], while Wolter et al. [8] indicated a positive effect of bilateral CI on postural control. However, Buhl et al. [9] concluded that CI surgery does not affect dynamic postural stability. Similar inconsistencies in the results can be found for the age of CI. However, the data regarding neuromuscular control are limited. For example, Louza et al. [10] and Mujdeci et al. [11] found that the age of participants with CI did not impact the postural balance performance, while Hocevar-Boltezar et al. [12] suggested that neuromuscular control can be significantly improved in children after CI. On the other hand, CI surgery can also be related to the incidence of post-surgery balance disorders, but they may result from factors other than surgery intervention [13, 14]. This can be mainly due to the activation of different compensatory auditory mechanisms, in which the strength of the kinesthetic, visual, and tactual senses, is related to several clinical and environmental factors [15].

Although CI has been the subject of previous studies, the data on postural control in late CI individuals in terms of different visual and auditory perceptions have been, to the authors’ knowledge, documented in only three studies [11, 16]. These studies allow, to some extent, for the identification of the variables related to postural control in CI individuals, but at the same time, they indicate the need for further and deeper analysis. Moreover, to the best of the authors’ knowledge, no study has evaluated postural balance in late lateral CI adolescents in terms of compensatory mechanisms of the auditory system. Given the above and the gap in the scientific literature, it seems justified to perform additional studies with the participation of individuals in the developmental period to evaluate the impact of late lateral CI surgery on neuromuscular control and to simultaneously identify determinants of the effectiveness of postural control. Therefore, the present study aimed to (1) assess the postural control in late lateral CI adolescents with different visual (eyes opened (EO)/eyes closed (EC)) and auditory (CI activated/deactivated) conditions and (2) build a theoretical model of postural control based on sensual compensatory mechanisms that are predominant in late CI individuals. It was hypothesized that kinesthetic sensation and exteroceptors of the superficial sensation are critical for neuromuscular control after late CI.

## Methods

### Study design

A quasi-experimental study protocol was used in this study in which the participants were theirs own controls in order to assess the postural stability performance with different visual and auditory perceptions.

### Participants

Twenty-seven adolescent students (nF = 13; nM = 14) with hearing loss from Polish special education schools and centers were purposively selected to participate in the study with the following inclusion criteria: (1) profound hearing loss in both ears, (2) hearing loss due to congenital or acquired impairment, (3) at least above 3 years old at the time of CI surgery, (4) lateral hearing apparatus (HA) in the opposite ear than CI, (5) at least 2 years after CI surgery, (6) females and males (aged 14–20 years old), (7) free from musculoskeletal or neuromuscular disorders, (8) satisfactory self-reported health status. The exclusion criteria were as follows: (1) participants with other impairments than hearing loss or mixed impairments, (2) males and females > 14 years old and/or < 20 years old, (3) the occurrence of musculoskeletal injury in the last three months, (4) no parental consent for participation in the study or withdrawal from the study.

In the majority of the study participants, hearing loss occurred at the age of up to 1 year (74%). In 19% of them, hearing loss was diagnosed at up to 2 years old, while only in 7%, it occurred after the age of 3 years. Similarly, the vast majority of both females (92%) and males (100%) were characterized by a congenital hearing impairment. Moreover, in 66.6% of study participants, CI surgery was performed after the age of 6 years, while 33.3% had it before the age of 6 years. Detailed characteristics of the study participants are presented in Table 1.

**Table 1:** Detailed characteristics of the study participants

Variables	SG Lateral CI and lateral HA (*n* = 27; nF = 13, nM = 14)	SG Females vs. Males		SG Females vs. Males
		Females (*n* = 13)	Males (*n* = 14)	*P* value
		Mean ± SD	Mean ± SD	
Age (years)	16.9 ± 1.8	17.0 ± 1.9	16.6 ± 1.7	*p* < 0.05
BH (cm)	168.8 ± 9.6	163.3 ± 7.4	175.0 ± 8.7	*p* > 0.05
BM (kg)	59.2 ± 10.7	57.6 ± 10.2	61.8 ± 11.8	*p* > 0.05
WC (cm)	74.7 ± 7.4	73.6 ± 8.2	75.9 ± 7.8	*p* > 0.05
HC (cm)	94.5 ± 8.5	91.4 ± 9.7	90.5 ± 8.2	*p* > 0.05
BMI	20.8 ± 3.5	21.6 ± 3.8	20.0 ± 3.1	*p* > 0.05
FM (%)	0.2 ± 0.1	26.2 ± 7.4	16.6 ± 6.0	*p* > 0.05
FM (kg)	12.9 ± 6.5	15.5 ± 6.6	10.9 ± 5.9	*p* > 0.05
FFM (kg)	46.0 ± 7.4	41.6 ± 5.1	50.8 ± 6.9	*p* > 0.05
FFM (%)	0.8 ± 0.1	0.7 ± 0.1	0.8 ± 0.1	*p* > 0.05
TBW (kg)	34.3 ± 6.3	30.5 ± 3.7	37.7 ± 5.6	*p* > 0.05
TBW (%)	0.6 ± 0.1	0.5 ± 0.1	0.6 ± 0.1	*p* > 0.05
CC – repose (cm)	86.3 ± 9.1	86.3 ± 9.1	83.3 ± 9.0	*p* > 0.05
CC – inspiration (cm)	89.5 ± 8.9	89.5 ± 8.9	87.6 ± 8.9	*p* > 0.05
CC – expiration (cm)	84.4 ± 9.1	84.4 ± 9.1	81.2 ± 8.9	*p* > 0.05
FL (cm)	40.8 ± 2.6	38.6 ± 1.2	43.1 ± 1.5	*p* > 0.05
Efficiency of the respiratory muscles	0.1 ± 0.1	0.01 ± 0.01	0.1 ± 0.0	*p* > 0.05
Age of CI surgery (years)	N/A	8.9 ± 3.9	9.2 ± 3.2	*p* < 0.05
Duration of using of the hearing device (years)	N/A	8.4 ± 3.2	9.2 ± 3.2	*p* < 0.05

SG – study group; CI – cochlear implantation; HA – hearing apparatus; n-total number of participants; nF- number of females; nM- number of males; SD – standard deviation; N/A – not applicable; BH – body height; BM – body mass; WC – waist circumference; HC – hips circumference; BMI – body mass index; FM – fat mass; FFM – fat free mass; TBW – total body water; CC – chest circumference; FL – foot length

The measurements were carried out in the laboratories of the Academy of Physical Education in Katowice, Poland, Institute of Sport Sciences, and in specially adapted rooms of the special education schools and centers that the study participants attended. Before starting the examinations, study participants were informed of the experimental procedures, benefits, and potential risks of the study and were instructed to maintain their normal dietary and sleeping habits for 24 h before the examination. Moreover, the participants were allowed to withdraw from the experiment at any time without giving a reason. Additionally, informed consent was obtained from all participants and from the legal guardians of the participants who were below 16 years of age. The research protocol was approved by the Bioethics Committee for Scientific Research of the Academy of Physical Education in Katowice, Poland (No. 9/2012) and met the ethical standards of the Declaration of Helsinki 2013.

### Procedures

All examinations were conducted in the morning (8–11 a.m.). At first, an interview in a form of a survey questionnaire was conducted to collect information about the participant’s health status and medical history of the hearing loss and cochlear implantation surgery (see Supplementary file). The questionnaire was fulfilled by study participant or his legal quardian (adequately to individual needs and participant’s age) and always with the presence of an experienced researcher (AK). Moreover, if study participant or legal guardian were deaf questions were asked in the sign language. Next, anthropometric examinations and postural stability testing were performed. All measurements were taken using the same research tools, temperature conditions, and the number and order of the measurements (including postural stability tests).

### Anthropometric measurements

The research protocol included the assessment of several qualities and indices of body build and posture. A wall-mounted stadiometer with a centimeter scale (accuracy of 1 cm) was used to measure body height (BH). The measurement of body mass (BM) was performed using a body composition analyser (TANITA TBF-300 M, accuracy of 0.1 kg). TANITA BC-420 MA Bioelectrical Impedance Analysis was used to calculate FM, FFM, and TBW. HC, WC, CC, and FL were assessed with an anthropometric tape, with an accuracy of 1 cm. A standard formula proposed by Zwierzchowska et al. [17] was used to calculate BMI. All measurements were performed by the same researcher (AK) with expertise in anthropometric evaluations.

### Postural stability testing and procedure

A force plate (Accu Gait AMTI, sampling frequency of 50 Hz, no filtering signal) with computer software (NetForce) was used in the study to assess the postural stability by evaluating three force components i.e., Fx, Fy, Fz, and the moment of force about the center of the force plate and around all three axes i.e., Mx, My, Mz. The center of feet pressure in the anteroposterior and lateral directions was calculated based on the values obtained for COP-Y, COP-AP (anteroposterior), and COP-X, COP-ML (lateral). Moreover, a methodology proposed by Zatsiorsky et al. [18] was used to assess the postural balance.

The postural stability testing protocol consisted of 4 trials that lasted 60 s each and were repeated twice. Each measurement was performed on a force plate to determine the velocity of the center of the foot pressure (vCOP) and the area of the ellipse (Area) by the same two researchers with expertise in postural stability (KS, AK) and with the presence of participant’s parents/legal guardians. The vCOP is known as a critical measure according to the evaluation of postural control and stability. Simultaneously, vCOP quantifies the speed at which the COP shifts within the base of support. Given that insights into the dynamic aspect of balance is provided. According to the present research, vCOP enables to detect the potencial deficiencies in the mechanisms of the postural control.

**Fig. 1:**
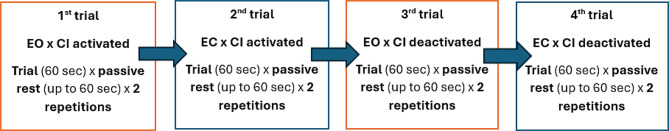
Schematic representation of the quasi-experimental design

Before the evaluation, study participants were informed (also in sign language) about the study protocol (see Fig. 1), and their main testing task was to keep the most motionless position as long as possible while standing on the force plate. The trial started with a verbal instruction ‘PLEASE ENTER THE PLATFORM’ and ended with ‘PLEASE GET DOWN FROM THE PLATFORM’, after which study participant took a short rest (up to 60 s, adjusted individually) and entered the force plate once again to begin the next trial. A hand-held timer was used by the same researcher (KS) to measure all breaks between study trials, while the time of all trials was recorded by computer software (NetForce). Because of difficulties with performing a full continuous trial, it was decided that only a selected part of the trial which was performed correctly (16 s) would be used in the statistical analyses. The study used the following 4 trials that were conducted twice, both with EO and EC.

1. Standing in the habitual position (arms along the trunk) with equal loading of the feet, looking straight ahead, EO, CI activated (trial time = 60 s).
2. Standing in the habitual position (arms along the trunk) with equal loading of the feet, looking straight ahead, EC, CI activated (trial time = 60 s).
3. Standing in the habitual position (arms along the trunk) with equal loading of the feet, looking straight ahead, EO, CI deactivated (trial time = 60 s).
4. Standing in the habitual position (arms along the trunk) with equal loading of the feet, looking straight ahead, EC, CI deactivated (trial time = 60 s).

### Statistical analysis

All statistical analyses were performed using the STATISTICA 13.3 computer software (TIBCO Software Inc., Tulska, OK, USA). The sample size was calculated using the following formula: fpc = sqrt((N-n)/(N-1)), where (i) fpc is the finite population correction factor, (ii) N is the population size, and (iii) n is the sample size. Kolmogorov-Smirnov test was used to verify distributions, homogeneity, means, and standard deviations (SD) with 95% confidence intervals of the anthropometric variables and participant’s characteristics (age, age of CI, duration of using the hearing device). An adjustment for the variables vCOP and Area and Tukey’s post-hock test were used to calculate the time of the measurement, which differed between the trials. The variety between vCOP (adjusted), Area (adjustment) and CI activated/deactivated was verified with the Mann-Whitney U Test with a Bonferroni correction. A multiple regression analysis with different visual and auditory conditions was performed in order to verify the impact of the selected clinical (age, gender, age of CI, hearing loss ethicology, participant’s age) and somatic (FL, BH, BM, WC, HC, CC, FM, FFT, TBW, BMI) variables on the postural stability. Correlations were evaluated as follows: trivial (0.0–0.09), small (0.10–0.29), moderate (0.30–0.49), large (0.50–0.69), very large (0.70–0.89), nearly perfect (0.90–0.99), and perfect (1.0) (Frost et al., 2020).

## Results

**Fig. 2:**
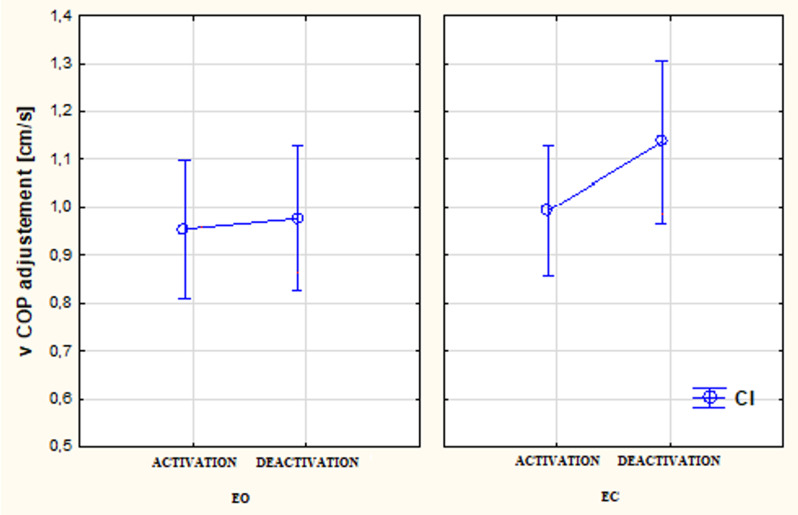
The differentiation of the vCOP (adjusted) (*p* < 0.05) in study participants with late lateral CI for the condition of EO x CI activated/deactivated and EC x CI activated/deactivated. Higher vCOP values indicate greater speeds of center of pressure movement, which may suggest reduced postural stability

Figure 2 shows the summary of the differentiation of the vCOP (adjusted) (*p* < 0.05) in study participants with late lateral CI with the condition of EO x CI activated/deactivated and EC x CI activated/deactivated. The Mann-Whitney U Test with Bonferroni correction revealed a significant growing tendency for the values of vCOP (adjusted) both in the conditions of CI activated and deactivated. Furthermore, a change of the vCOP (adjusted) with different visual conditions (EO/EC) was found but statistical analysis did not confirm the statistical significance (*p* > 0.05).

**Fig. 3:**
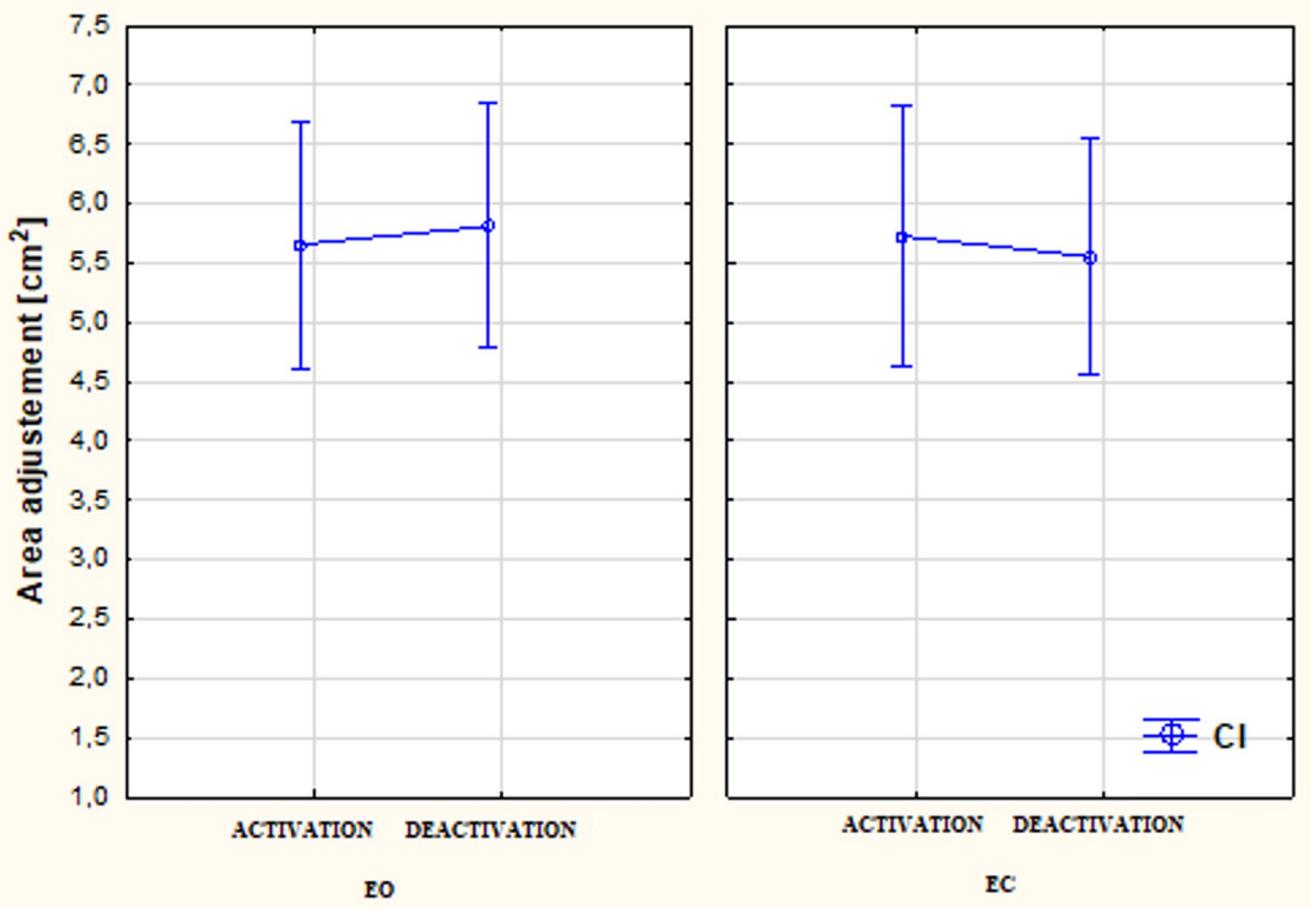
The differentiation of the Area (adjusted) (*p* < 0.05) in study participants with late lateral CI with the condition of EO x CI activated/deactivated and EC x CI activated/deactivated

Figure 3 presents the differentiation of the Area (adjusted) (*p* < 0.05) in study participants with late lateral CI with the condition of EO x CI activated/deactivated and EC x CI activated/deactivated. No statistically significant relationships were noted between the range of the displacement of feet pressure (Area) and both the visual (EO/EC) and auditory (CI activated/CI deactivated) conditions.

**Fig. 4:**
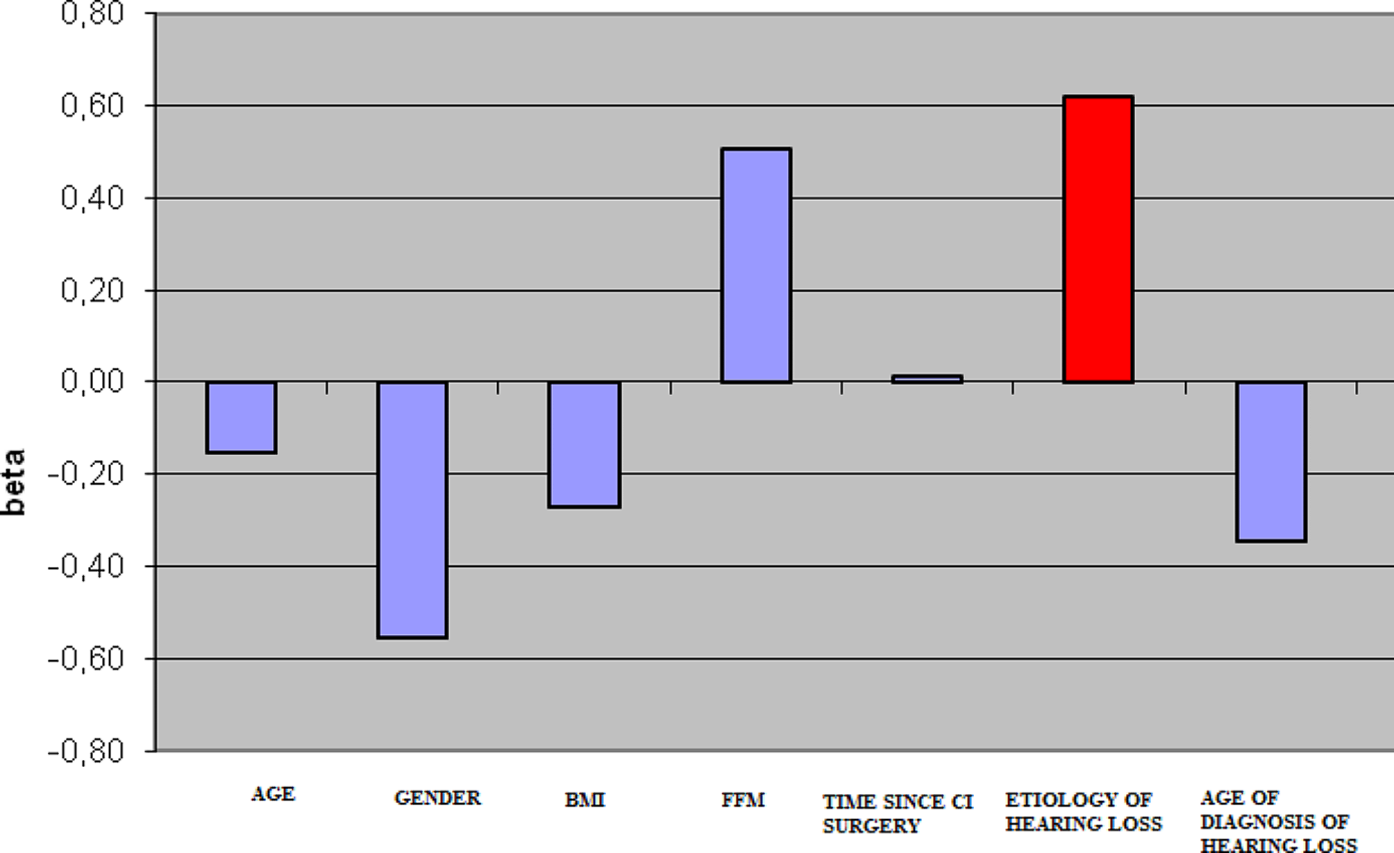
The differentiation of the vCOP(adjusted) (*p* < 0.05) in study participants with late lateral CI with the condition of EO x CI activated/deactivated

The differentiation of the vCOP(adjusted) (*p* < 0.05) in study participants with late lateral CI with the condition of EO x CI activated/deactivated is shown in Fig. 4. The multiple regression analysis indicated that hearing loss etiology was statistically significantly related to the values of vCOP (see Fig. 4). It was found that the greater the values of the velocity of vCOP, the greater the impact of the hearing loss etiology was within the conditions of EO and CI activated/deactivated (*p* < 0.01). Moreover, this dependency did not occur with the conditions of EC and CI activated/deactivated (*p* > 0.05).

**Fig. 5:**
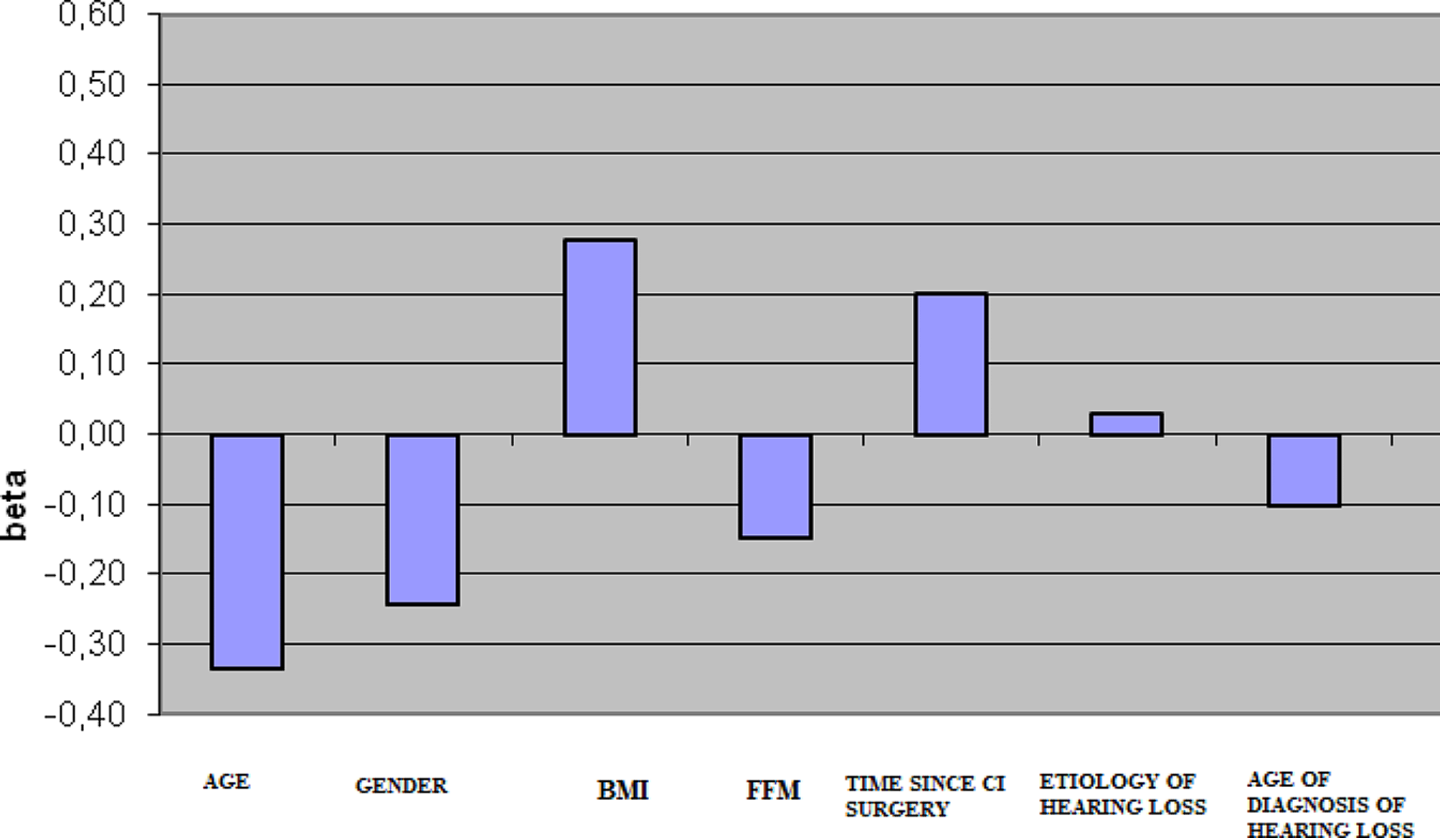
The differentiation of the Area (adjusted) (*p* < 0.05) in study participants with late lateral CI with the condition of EO x CI activated/deactivated

The summary of the differentiation of the Area (adjusted) (*p* < 0.05) in study participants with late lateral CI with the condition of EO x CI activated/deactivated is shown in Fig. 5. It was found that no statistically significant differences occurred for changes in the Area due to different visual (EO/EC) and auditory (CI activated/deactivated) conditions.

**Fig. 6:**
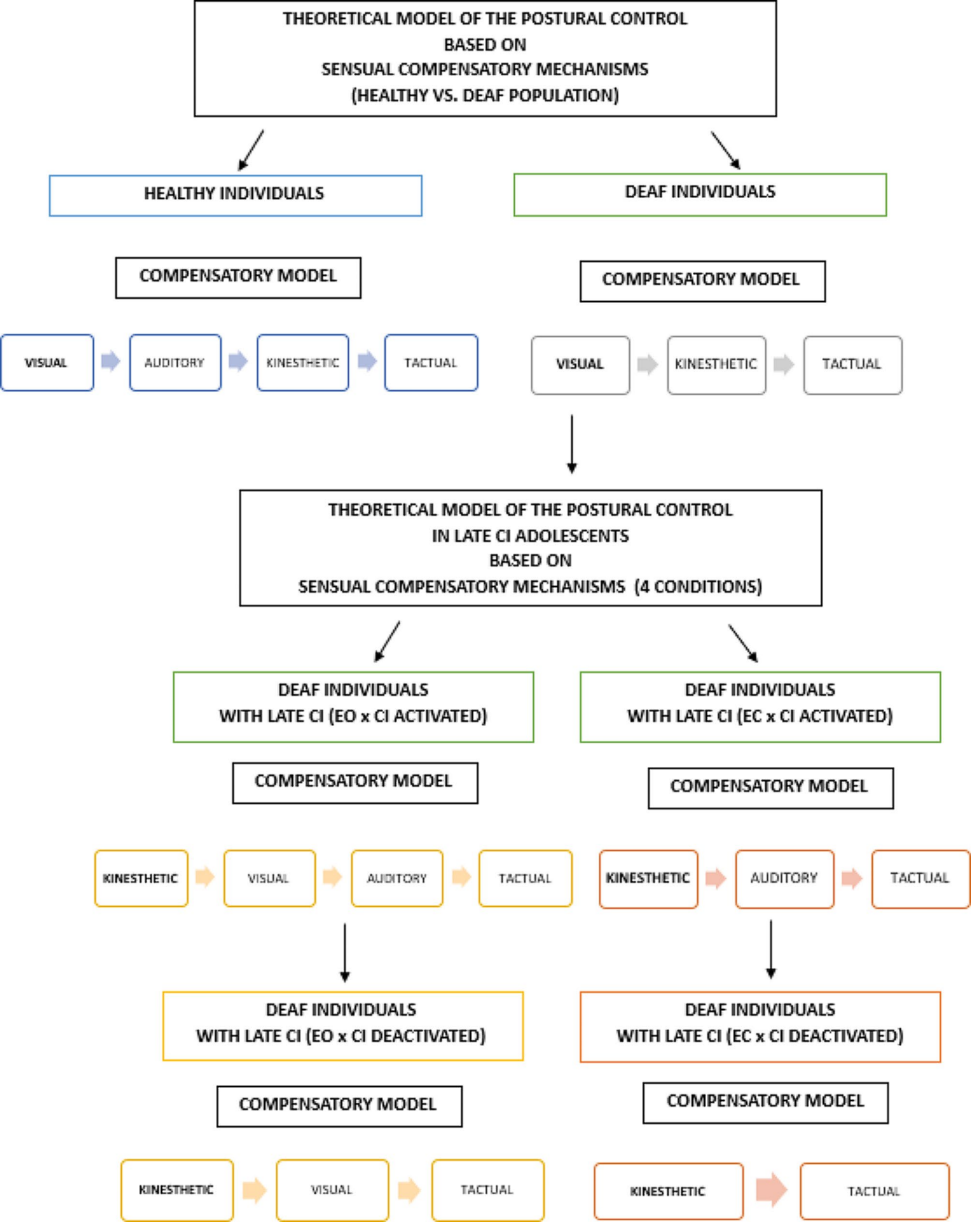
A theoretical model of the postural control in healthy and deaf population and in late CI adolescents based on sensual compensatory mechanisms

A theoretical model of postural control in hearing and deaf population based on sensual compensatory mechanisms is presented in Fig. 6. The flow of the diagram includes division on the hearing individuals and those who are deaf, simultaneously indicating the primary sense that seems to be primarly to maintain the postural control in the abovementioned populations. Moreover, the diagram was expanded by the results of the present study, including the crucial senses that seem to be related to the postural control in adolescents who use CI an HA in everyday life. Given the obtained results of the vCOP and Area, it was found that the neuromuscular control with the condition of EC x CI deactivated seems to be based on the kinesthetic-tactual compensatory model.

## Discussion

The data on the sensual compensatory mechanisms in the deaf population remain limited. Therefore, this study aimed to evaluate postural control in late lateral CI adolescents within different visual and auditory perceptions and to simultaneously build a theoretical model of compensatory mechanisms related to the reception of auditory stimuli in late CI individuals. As was assumed in the initial hypothesis, this study found that within the conditions of EC x CI deactivated, neuromuscular control is regulated mainly by the kinesthetic sensation and exteroceptors of the superficial sensation (kinesthetic-tactual compensatory model). Thus, this result indicates that kinesthetic sensation has a significant impact on the magnitude of vCOP and Area in deaf individuals with late CI (see Fig. 6). This also suggests that to improve postural balance performance in this population, it is recommended that the patients perform physical activity tasks, especially to develop core muscles, which are based on direct stimulation and rotational stability.

To the best of the authors’ knowledge, although many studies have evaluated postural balance in different perceptual and/or visual conditions [10, 12, 19], only three studies have directly compared their impact on postural control performance in late CI individuals [11, 16, 21]. Although in the case of enhancement of the neuromuscular control, the most commonly recommended intervention is currently early CI [20], no study to date has considered the effect of varied visual and auditory conditions on the vCOP and Area in adolescents with late lateral implantation.

The results of our study are partially in line with the findings of Suarez et al. [21] who indicated the primary role of visual and somatosensory information for maintaining postural control in deaf children with unilateral CI, as they found an increase in the values of vCOP for the condition of EC x CI activated, which was also confirmed by our study. However, our analysis suggested that kinesthetic sensitivity seems to be primary compared to visual stimulus (see Fig. 6). This thesis can be explained by the different sensory compensatory mechanisms models that are activated due to different visual and auditory perceptions. Furthermore, based on the comparison of the values of vCOP and Area that differ due to testing conditions, it seems that postural control is dependent primarily on kinesthetic sensation.

On the other hand, information from stimulus is not the only source that impacts the postural balance as our regression analysis found that vCOP can be predicted by the etiology of hearing loss (congenital/acquired). This was also confirmed in a study by Cushing et al. [22], who found that vestibular and balance dysfunction in unilateral CI children is highly dependent on deafness etiology. However, Suearez et al. [21] concluded that deafness etiology is not related to the reception of the somatosensory information to maintain postural control, simultaneously indicating the predominant role of visual and somatosensory systems in regulating postural balance. The inconsistency in the cited studies can be explained by the neurophysiological factors [15] arising from the activation of different sensory compensatory mechanisms models, which are mainly related to (a) time of disability (years), (b) etiology of hearing loss (congenital/acquired), and (c) age at hearing loss.

Saurez et al. [21] examined the problems of sensory compensatory mechanisms and found an increase in the values of vCOP for the absence of visual perception and CI activated. Similar results were obtained in our study. Furthermore, the increase in the vCOP was even higher within the condition of EC x CI deactivated, while the values of Area showed a decreasing tendency. Thus the findings of our study can in some way explain the diversity of the values of vCOP and Area that could be a consequence of an increase in the foot pressure by activation of the kinesthetic-tactual compensatory model when excluding visual perception (see Fig. 6). However, as indicated by Swamy Suman et al. [23] central vestibular compensation should also be taken into consideration as it’s related to reduction of the vestibular symptoms and can also contribute to improvement of the balance in individuals with vestibular disfunction. Nevertheless, to fully understand the complexity of the sensory compensatory models in the deaf population, including people with CI, further studies are needed, also to assess and compare different age groups and hearing devices i.e., bilateral CI and auditory apparatus. This may enable to explain the intrinsic differences that can possibly occure in the postural balance between adolescents with CI and those who use only HA. Such attitude will also allow for general interference about the main model of sensual compensatory mechanisms, that can be diverse due to hearing devices (CI/HA) that are used by individuals with hearing impairments.

### Limitations

The present study has several limitations that need to be addressed. First of all, we did not include a control group of healthy non-deaf adolescents that would enable us to compare the diversity of the vCOP and Area. Thus, our results cannot be generalized to the healthy population. However, the studied group was homogeneous and almost perfectly equal in terms of the number of males (*n* = 14) and females (*n* = 13), which is the strength of the present paper. Secondly, the study was performed on inactive adolescents, thus the results may differ in active unilaterally implanted populations. Lastly, we did not include other study groups of bilateral CI adolescents and adolescents with bilateral auditory apparatus, which would allow for better understanding of the complexity of the neuromuscular responses to different visual and auditory perceptions.

## Conclusions

The results of this study indicated that kinesthetic sensation and exteroceptors of the superficial sensation seem to be the predominant source of information to maintain postural control in late CI adolescents, regardless of the visual and auditory conditions. Moreover, the etiology of hearing loss (congenital/acquired) can be a predictor of the values of the vCOP. The practical implication of the present study is that direct stimulation in the form of core muscle exercises and rotational stability tasks should be recommended to improve neuromuscular control.

### Electronic supplementary material

Below is the link to the electronic supplementary material.

Supplementary Material 1

## Data Availability

The data collected and analyzed during the current study are available from the corresponding author on reasonable request.

## Abbreviations

BH: Body height
BM: Body mass
BMI: Body mass index
CC: Chest circumference
CI: Cochlear implantation
EC: Eyes closed
EO: Eyes opened
FFM: Fat free mass
FL: Foot length
FM: Fat mass
HA: Hearing apparatus
HC: Hips circumference
N/A: Not applicable
nF: Number of females
nM: Number of males
n: Total number of participants
SD: Standard deviation
SG: Study group
TBW: Total body water
vCOP: Velocity of the center of the foot pressure
WC: Waist circumference

## References

[1] Louza J, Rösel C, Gürkov R, Krause E, Ihler F. Influence of Cochlear Implantation on Postural Control and Risk of Falls. Audiol Neurootol. 2019;24(5):245–52. 10.1159/000503165. 31639802 10.1159/000503165

[2] Coordes A, Basta D, Götze R, Scholz S, Seidl RO, Ernst A, Todt I. Sound-induced vertigo after cochlear implantation. Otol Neurotol. 2012;33(3):335 – 42. 10.1097/MAO.0b013e318245cee3. 10.1097/MAO.0b013e318245cee3 22334157

[3] Batuecas-Caletrio A, Klumpp M, Santacruz-Ruiz S, Benito Gonzalez F, Gonzalez Sánchez E, Arriaga M. Vestibular function in cochlear implantation: correlating objectiveness and subjectiveness. Laryngoscope. 2015;125(10):2371–5. 10.1002/lary.25299. 25891786 10.1002/lary.25299

[4] Todt I, Rademacher G, Mutze S, Ramalingam R, Wolter S, Mittmann P, Wagner J, Ernst A. Relationship between intracochlear electrode position and tinnitus in cochlear implantees. Acta Otolaryngol. 2015;135(8):781–5. 10.3109/00016489.2015.1024332. 25812721 10.3109/00016489.2015.1024332

[5] Keshner EA, Cohen H. Current concepts of the vestibular system reviewed: 1. The role of the vestibulospinal system in postural control. Am J Occup Ther. 1989;43(5):320–30. 10.5014/ajot.43.5.320. 10.5014/ajot.43.5.320 2655458

[6] Kelly A, Liu Z, Leonard S, Toner F, Adams M, Toner J. Balance in children following cochlear implantation. Cochlear Implants Int. 2018;19(1):22–5. 10.1080/14670100.2017.1379180. 28946841 10.1080/14670100.2017.1379180

[7] Miwa T, Minoda R, Matsuyoshi H, Takeda H. The effect of cochlear implants on vestibular-evoked myogenic potential responses and postural stability. Auris Nasus Larynx. 2019;46(1):50–7. 10.1016/j.anl.2018.06.002. 29935892 10.1016/j.anl.2018.06.002

[8] Wolter NE, Gordon KA, Campos J, Vilchez Madrigal LD, Papsin BC, Cushing SL. Impact of the sensory environment on balance in children with bilateral cochleovestibular loss. Hear Res. 2021;400:108134. 10.1016/j.heares.2020.108134. 33310565 10.1016/j.heares.2020.108134

[9] Buhl C, Artemiev D, Pfiffner F, Swanenburg J, Veraguth D, Roosli C, Huber A, Dalbert A. Dynamic Postural Stability and Hearing Preservation after Cochlear Implantation. Audiol Neurootol. 2018;23(4):222–8. 10.1159/000494247. 30428457 10.1159/000494247

[10] Louza J, Klappert CL, Ledderose G, Gürkov R, Krause E. Cochlear Implant surgery and the risk of Falls in an Adult Population. Otol Neurotol. 2018;39(2):e74–9. 10.1097/MAO.0000000000001656. 29315181 10.1097/MAO.0000000000001656

[11] Mujdeci B, Önder S, Alluşoğlu S, Boynuegri S, Kum O, Atan D. The effects of age at cochlear implantation on balance in children: a pilot study. Int J Artif Organs. 2021;44(6):440–5. 10.1177/0391398820967367. 33143530 10.1177/0391398820967367

[12] Hocevar-Boltezar I, Boltezar M, Zargi M. The influence of cochlear implantation on vowel articulation. Wien Klin Wochenschr. 2008;120(7–8):228–33. 10.1007/s00508-008-0944-2. 18500598 10.1007/s00508-008-0944-2

[13] Colin V, Bertholon P, Roy S, Karkas A. Impact of cochlear implantation on peripheral vestibular function in adults. Eur Ann Otorhinolaryngol Head Neck Dis. 2018;135(6):417–20. 10.1016/j.anorl.2018.10.007. 30431000 10.1016/j.anorl.2018.10.007

[14] Wiszomirska I, Zdrodowska A, Tacikowska G, Sosna M, Kaczmarczyk K, Skarżyński H. Does cochlear implantation influence postural stability in patients with hearing loss? Gait Posture. 2019;74:40–4. 10.1016/j.gaitpost.2019.08.013. 31442821 10.1016/j.gaitpost.2019.08.013

[15] Zwierzchowska A, Krużyńska A. Hearing impairment published in: Zwierzchowska, A., Sosnowska-Wieczorek, I., Morawski, K. The child with hearing impairment. Implications for theory and practice. (2019). Academy of Physical Education in Katowice, Poland, ISBN- 978-83-66308-01-5, pp. 53–74.

[16] Suarez A, Ferreira E, Garcia Pintos B, Arocena S, Suarez H. Postural control characterization according to age and auditory input in cochlear implant users. Cochlear Implants Int. 2021;22(1):29–34. 10.1080/14670100.2020.1813996. 32900289 10.1080/14670100.2020.1813996

[17] Zwierzchowska A, Grabara M, Palica D, Zając A. BMI and BAI as markers of obesity in a caucasian population. Obes Facts. 2013;6(6):507–11. 10.1159/000356402. 24217471 10.1159/000356402 PMC5644723

[18] Zatsiorsky VM, Duarte M. Instant equilibrium point and its migration in standing tasks: rambling and trembling components of the stabilogram. Motor Control. 1999;3(1):28–38. 10.1123/mcj.3.1.28. 9924099 10.1123/mcj.3.1.28

[19] Čakrt O, Slabý K, Kučerová K, Balatková Z, Jeřábek J, Bouček J. Subjective visual vertical and postural control in patients following cochlear implantation. J Vestib Res. 2023;10. 10.3233/VES-220136. 10.3233/VES-220136 37574747

[20] Purcell PL, Deep NL, Waltzman SB, Roland JT Jr, Cushing SL, Papsin BC, Gordon KA. Cochlear implantation in infants: why and how. Trends Hear. 2021;25:23312165211031751. 10.1177/23312165211031751. 34281434 10.1177/23312165211031751 PMC8295935

[21] Suarez H, Angeli S, Suarez A, Rosales B, Carrera X, Alonso R. Balance sensory organization in children with profound hearing loss and cochlear implants. Int J Pediatr Otorhinolaryngol. 71(4)(2007)629 – 37. 10.1016/j.ijporl.2006.12.014. 10.1016/j.ijporl.2006.12.014 17275927

[22] Cushing SL, Papsin BC, Rutka JA, James AL, Gordon KA. Evidence of vestibular and balance dysfunction in children with profound sensorineural hearing loss using cochlear implants. Laryngoscope. 118(10)(2008)1814-23. 10.1097/MLG.0b013e31817fadfa. 10.1097/MLG.0b013e31817fadfa 18758383

[23] Swamy Suman N, Kumar Rajasekaran A, Yuvaraj P, Pruthi N, Thennarasu K. Measure of central vestibular compensation: a review. J Int Adv Otol. 2022;18(5):441–6. 10.5152/iao.2022.21207. 35971266 10.5152/iao.2022.21207 PMC9524397

