# Supplementary material for: Postural stability at activation and deactivation of the cochlear implant in adolescents with late lateral implantations: a quasi-experiment
Source: BMC Sports Sci Med Rehabil. 2024 Jul 21;16:159. doi: 10.1186/s13102-024-00950-1 (PMC11265106; doi:10.1186/s13102-024-00950-1)
Supplement: Supplementary file 1 — Supplementary Material 1 [file 13102_2024_950_MOESM1_ESM.docx]

**SURVEY QUESTIONNAIRE**

*The research team would like to fulfill the following questions by writing ‘X’ in the □ or by writing the answer in the empty places. All data obtained during the research will be collected according to the rules and regulations of protecting the personal data. Only the project’s head will know which data are related to you. Personal data will be treated confidentially and would not be able to be identified in any publication prepared as the part of this research. The obtained results will be used only for scientific aims.*

**METRICS:**

1. Name and surname ____________________________________
2. Date of birth ____________________________________
3. Lateralization:

- eye ______________________________
- upper limb (arm) ______________________________
- lower limb (leg) ______________________________

1. Do you have a cochlear implant (CI) :

□ year and place in which you have CI surgery: ____________________________________________________

1. CI placed on the:

- Right side ______________________________
- Left side ______________________________

1. Where do you live (please add the city name) :

□ in a big city (over 100 thousand citizens)

□ in a small city (up to 100 thousand citizens)

□ in the countryside

1. Who is deaf in your family:

□ mother □ father

□ brother □ sister

□ grandfather □ grandmother

□ other, who ­­­­­­­­­­­­­­­­­­­­? ­­___________________

1. When have you been diagnosed with hearing defect? Please add the accurate year of your life. ____________­­­­­­­­­­­­­­­­­­­­­­­­_________________________________________
2. Please cross the reason for you hearing loss:

□ congenital or genetic defect (my parents and/or grandparents are deaf)

□ infectious disease occured in mother during pregnency (e.g,.:

floret)

□ hearing defect as a reason of ototoxic drugs

(e.g.,.: gentamicyn, streptomycin)

□ ear injury (mechanical)

□ acoustic injury (loud noise)

□ ear infection (ear illness)

□ it is unknown why I can not hear

□ other reasons ____________________________________________________________________________________________________________

1. What kind of hearing defect do you have?

□ conductive

□ mixed (conductive-processing)

□ processing

□ central

1. Your hearing loss in dB:
2. *Before CI:*
3. Left ear __________ dB Right ear ________ dB
4. *After CI:*
5. Left ear __________ dB Right ear _________ dB
6. What changes have been observedin your daily routine after CI:

□ I have started to hear different, new kinds of sound

□ I have improved my speaking, I’m speaking better

□ I have stopped beeing dependent from the others, I am more independent e.g., in a shop, at post office, at school

□ Nothing has changed

1. How is your daily contact with your family (parents, grandparents, aunt, uncle) after CI?

□ Better

□ Worse

□ Nothing has changed

1. How is your daily contact with your sibling?

□ Better

□ Worse

□ Nothing has changed

□ I do not have a sibling

1. How is your daily contact with your friends and colleagues at school?

□ Better

□ Worse

□ Nothing has changed

1. Have CI improved your understanding during lessons (do you get better marks)?

□ Yes □ No

□ Only on some subjects.: _______________________________________________________

1. Have you wanted to have CI surgery ?

□ Yes

□ No

1. I take off my CI during P.E. lesson:

□ Yes

□ No

□ I do not participate in P.E. lesson

1. Please mark/write your favourite weekly forms of physical activity during week (minimum 3xweek):

□ football

□ running

□ swimming

□ bike riding

□ individual exercises at home, fitness

□ others (which)

20. What was your way of communication ?

1. *Before CI* b) *After CI*

□ sign language □ sign language

□ gestures □ gestures

□ mounth reading □ mounth reading

□ drawings □ drawings

□ writing □ writing

□ others ____________________ □ others _____________
